# The link between hydrotropism and phototropism in Arabidopsis roots

**DOI:** 10.1093/jxb/erad293

**Published:** 2023-09-12

**Authors:** Arpita Yadav

**Affiliations:** Biology Department, University of Massachusetts, Amherst, MA 01003, USA

**Keywords:** Auxin, gravitropism, hydrotropism, phototropism, roots

## Abstract

This article comments on:

Pang L, Kobayashi A, Atsumi Y, Miyazawa Y, Fujii N, Dietrich D, Bennett MJ, Takahashi H. 2023. MIZU-KUSSEI1 (MIZ1) and GNOM/MIZ2 control not only positive hydrotropism but also phototropism in Arabidopsis roots. Journal of Experimental Botany 74, 5026–5038.

**When exposed to one-sided blue light, the roots of plants such as *Arabidopsis thaliana* exhibit negative phototropism and curve away from the light. The genes *MIZU-KUSSEI1* (*MIZ1*) and *GNOM/MIZ2* play important roles in positive hydrotropism, the process by which roots grow in a direction towards water. Intriguingly, mutations in these genes cause a dramatic loss of phototropism. Pang *et al*. (2023) found out that the same tissues in Arabidopsis roots involved in MIZ1- and GNOM/MIZ2-regulated hydrotropism are also required for phototropism.**

While most animals move around to meet their needs, plants display variable growth movements to position their organs so that they can thrive. To move their organs, plants have evolved various tropisms that determine the direction and magnitude of growth in response to an environmental gradient, such as gravity, light, water, temperature, touch, and even pathogens (Cassab *et al.*, 2013). Tropisms enable plants to adjust their growth direction in response to environmental changes.

Tropic processes are divided into three steps: perception, signal transduction, and growth response. These steps are probably best understood for roots responding to gravity. Perception occurs as gravity moves heavy starch grains in cells of the central root cap to the ‘earthward’ face of the cell. Signal transduction integrates the signal provoked by these starch grains across the root cap, allowing the direction of gravity to be inferred and compared with the suitable direction. Finally, growth response occurs as the signal transduction output is communicated to the root elongation zone. To understand any tropism, we need to understand how the relevant gradient is perceived and how that information is converted into differential growth.

Here, Pang *et al*. (2023) reveal a surprising commonality in the responses of roots to light (phototropism) and water (hydro­tropism). Arabidopsis roots respond to a blue light gradient by bending away from the brightest light; in contrast, roots respond to a gradient of moisture by bending towards greater moisture. These growth responses seem clearly adaptive to provide adequate water for the plant and avoid getting dried out by exposure to sunlight shining into the rhizosphere through ground disturbances. Tropisms depend on various parts of the root. Roots are zoned, with the growing part of the root partitioned into cap, meristem, and elongation zone; the root is also divided into tissues: columella and lateral root cap in the cap; epidermis, cortex, endodermis as the cell layer separating root cortex and stele, and stele in the root body. The stele is divided into pericycle, xylem, phloem, and parenchymatic cells.

The authors investigated the function of the proteins MIZ1 and MIZ2, which were previously identified in ahydrotropic mutants unable to respond to moisture gradients. In Arabidopsis roots, the authors compare the functions of these proteins in hydrotropism and phototropism. To study these proteins, they used genomic analysis, imaging techniques, and physiological experiments.

MIZ1 encodes an endoplasmic reticulum (ER)-associated membrane protein that is known to negatively regulate the ER Ca^2+^-ATPase (Shkolnik *et al.,* 2018). Expression of the fluorescent reporter MIZ1–green fluorescent protein (GFP) driven by the putative *MIZ1* promoter shows that *MIZ1* is expressed strongly in the root cap and in cortex cells throughout the meristem and into the elongation zone, and to a lesser extent in the epidermis and stele (Yamazaki *et al.,* 2012; Moriwaki *et al.,* 2013). The intensity and localization of *MIZ1–GFP* expression do not change during a hydrotropic response. *MIZ1* expression in the root cortex is sufficient to rescue the hydrotropism in *miz1* mutants. In contrast, expressing *MIZ1* in the root cap was ineffective in rescuing the hydrotropic response in *miz1* roots (Dietrich *et al.,* 2017). Interestingly, *MIZ2* was shown to be an allele of *GNOM* which encodes a guanine nucleotide exchange factor for ADP ribosylation factor (ARF-GEF) (Miyazawa *et al.,* 2009). One of the most well-studied roles of GNOM is in controlling the trafficking of PIN auxin efflux carrier proteins (Kleine-Vehn *et al.,* 2008; see Box 1 for details about MIZ1 and GNOM).

**Box 1. More information about MIZ1 and GNOM**

**MIZ1**

MIZ1 was found in an EMS mutant screen in Arabidopsis for altered hydrotropic response. The full mutant name, *mizu-kussei* (*miz*), combines the words for ‘water’ (*mizu*) and ‘tropism’ (*kussei*) in Japanese. Roots of *miz1* mutants, which are impaired in hydrotropism, have wild-type-like gravitropism, elongation growth, and root tip anatomy, but impaired phototropism (Kobayashi *et al.,* 2007). The phenotypic changes result from a single recessive mutation in *MIZ1*, At2g41660, which encodes a protein containing a domain (the MIZ domain) that is highly conserved among terrestrial plants such as rice and moss. Unfortunately, MIZ1 is a protein with an uncharacterized function; all we know about it is that it contains a conserved region called the DUF617 domain. The discovery of DUF617 domain-containing homologs in rice and *Physcomitrella patens*, but not in algae, suggests that land plants acquired MIZ1 function in the course of evolution (Kobayashi *et al.,* 2007). MIZ1 is a soluble protein associated with the cytosolic side of the ER membrane (Yamazaki *et al.,* 2012). Both abscisic acid (ABA) and blue light can increase *MIZ1* expression (Moriwaki *et al.,* 2012). MIZ1 itself appears to affect auxin accumulation, as free indole-3-acetic acid (IAA) concentrations increase in *miz1* roots and decrease in *MIZ1*-overexpressing roots (Moriwaki *et al.,* 2011).

**GNOM**

GNOM was found in an EMS-induced screen for developmental mutants. *gnom* mutants have a striking phenotype, a ball-shaped embryo that dies soon after germination, and they largely fail to form root meristem and cotyledon primordia (Mayer *et al.,* 1993). Weaker alleles allow development to varying extents. Most anomalies linked to GNOM loss of function pertain to modified auxin transport, and GNOM function is necessary for localizing PIN-family auxin efflux carriers (Steinmann *et al.,* 1999; Geldner *et al.,* 2003). GNOM is also known to be involved in cold stress response (Ashraf and Rahman, 2019). The gene comprises a central catalytic domain, similar to the secretory protein Sec7p of yeast, known as the SEC7 domain. Arabidopsis GNOM cDNA partially complemented a mutant in the yeast homolog. *GNOM* encodes a guanine nucleotide exchange factor that specifically targets ADP ribosylation factor (ARF-GEF) in small GTP-binding proteins. The exchange of GDP to GTP is a crucial step in the activation of ARF GTPases, which in turn facilitate vesicle formation. As a result, GNOM regulates vesicle trafficking. The *miz2* allele of *gnom* is noteworthy as it confers ahydrotropic growth but not agravitropic growth, meaning that the *miz2* mutants are unable to grow towards higher water potential but at the same time are able to sense gravity, indicating that vesicular trafficking plays different roles in hydrotropism and gravitropism in roots.

The objective of the work of Pang *et al.* (2023) was to investigate the involvement of MIZ1 and GNOM (MIZ2) in phototropism. The authors show that as in hydrotropism, expressing *MIZ1* specifically in the cortex restores full responsiveness to blue light in *miz1* mutants. They examined the phototropic response of plants expressing *MIZ1–GFP* under the control of different promoters such as *ROOT CLAVATA HOMOLOG1* (*RCH1*), *SOMBRERO* (*SMB*), or *SCARECROW* (*SCR*), and found that the roots bend slightly away from blue light and had phototropic curvature compared with untransformed *miz1* mutants. However, when *MIZ1–GFP* was expressed under the cortex promoter in *miz1*, the mutants exhibited phototropic response like the wild type. The authors also ruled out the possibility of involvement of epidermis cells in the phototropic response of roots.

Expressing *GNOM–GFP* in the *miz2* background under different tissue-specific promoters showed that while non-transformed *miz2* roots exhibited no hydrotropic response, the roots of wild-type and *GNOMpro:GNOM-GFP* transgenic plants did. The *GNOMpro:GNOM-GFP* construct completely restored the hydrotropic deficit of *miz2* mutant roots. *SMBpro:GNOM-GFP* transgenic roots phenocopied *miz2* mutant roots, indicating that GNOM is not necessary for hydrotropism in the root cap. Compared with wild-type roots, the roots of *SCRpro:GNOM-GFP* transgenic plants displayed a minor hydrotropic response, indicating that the root cap and endodermal cells are not essential for the function of GNOM in hydrotropism. In contrast, the transgenic plants with *RCH1pro:GNOM-GFP*, *WERpro:GNOM-GFP*, *Co2pro:GNOM-GFP*, and *SHRpro:GNOM-GFP* roots bowed toward the water-rich agar. This shows that *GNOM* expression in the epidermis, cortex, or stele is necessary for hydrotropism.

Laser ablation of the root cap and meristem in transgenic lines had no influence on hydrotropism recovery. In addition, the phototropic responses of transgenic plants expressing *GNOM–GFP* in various tissues were investigated. Transgenic *GNOMpro:GNOM-GFP* roots showed perfect phototropism, identical to wild-type roots, but transgenic *SMBpro:GNOM-GFP* and *SCRpro:GNOM-GFP* roots showed reduced phototropism. However, the roots of transgenic plants expressing *RCH1pro:GNOM-GFP*, *WERpro:GNOM-GFP*, *Co2pro:GNOM-GFP*, and *SHRpro:GNOM-GFP* demonstrated a complete phototropic response, identical to wild-type roots. This showed that *GNOM* expression in the epidermis, cortex, or stele is essential for Arabidopsis root hydrotropism and phototropism.

Overall, the study shows that MIZ1 acts in the cortex of the Arabidopsis root elongation zone not only in hydrotropism but also in phototropism (Fig. 1). Likewise, GNOM functions in both tropisms but this protein appears able to act in either the epidermis, the cortex, or the stele. These results imply that the two tropisms partially engage homologous pathways.

**Fig. 1.**
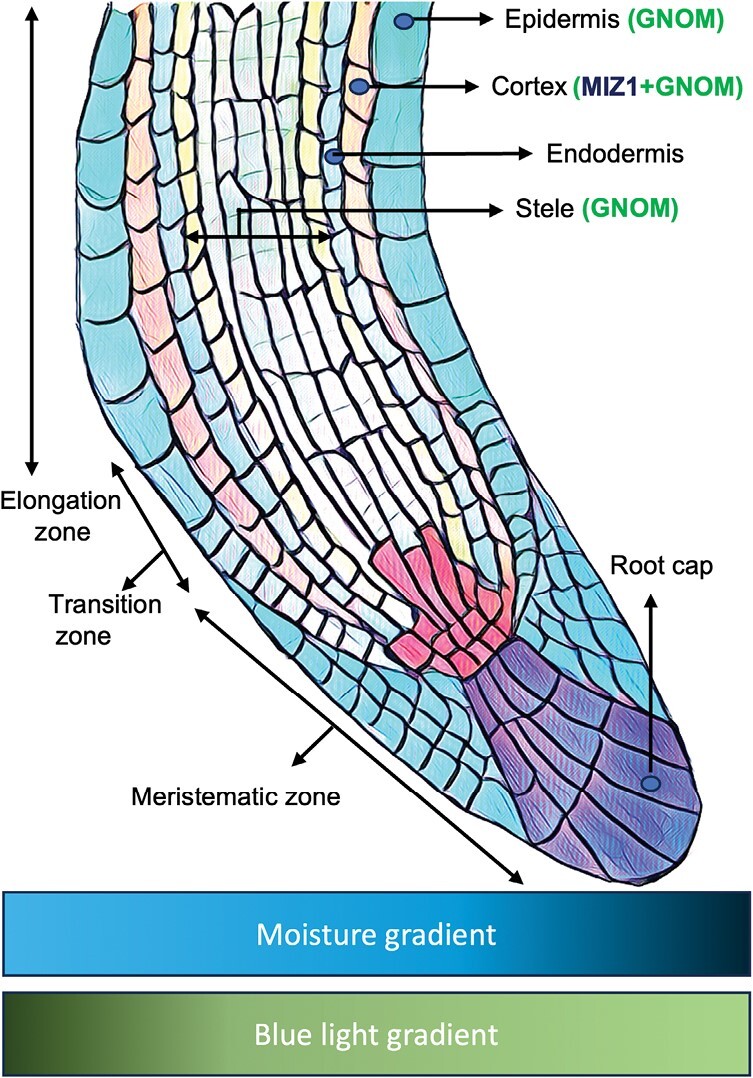
Schematic representation of the longitudinal cross-section of the root apex bending towards higher moisture content (hydrotropism) and away from the blue light signal (phototropism). The root can be divided into different developmental zones (meristematic zone, transition zone, and elongation zone) and the function of MIZ1 and GNOM are restricted to certain cell layers in the elongation zone: GNOM functions in the epidermis, cortex, and stele, whereas MIZ1 functions in the cortex during hydrotropism and phototropism.

## The way forward

This study adds to our understanding of how plants use information from their surroundings to tune their growth and development. The discovery of MIZ1 and GNOM as key players in both hydrotropism and phototropism sheds light on how various environmental stimuli are coordinated during root growth. The finding that phototropism and hydrotropism both require these proteins in the same tissue supports the hypothesis that the signaling pathways of the two root growth processes evolved from a common ancestral response and still share components. The question arises of how the root would respond when exposed to a gradient of blue light in parallel with a gradient in moisture. Phototropin (phot1) is the primary blue light receptor in roots. The N-terminus of phot1 protein has two similar domains called LOV domains which are involved in sensing light, oxygen, and voltage. The LOV domains interact with flavin upon blue light perception and undergo an autophosphorylation reaction eventually leading to bending of roots. However, the cellular signaling events following the autophosphorylation reaction are largely unknown. Given that roots perceive blue light via phototropin, one also wonders whether the already versatile LOV domain of phot1 might have evolved to respond to water potential as well.
